# New Jersey

**Published:** 1896-07

**Authors:** 


### CONTROL WORK.

*New Jersey.* Thoroughbred cattle to the value of six hundred dollars were recently destroyed at Stewartsville by the New Jersey Tuberculosis Commission.

